# Characterization and Monitoring of Antigen-Responsive T Cell Clones Using T Cell Receptor Gene Expression Analysis

**DOI:** 10.3389/fimmu.2020.609624

**Published:** 2021-02-19

**Authors:** Sabrina Pollastro, Marie de Bourayne, Giulia Balzaretti, Aldo Jongejan, Barbera D. C. van Schaik, Ilse T. G. Niewold, Antoine H. C. van Kampen, Bernard Maillère, Niek de Vries

**Affiliations:** ^1^Department of Clinical Immunology & Rheumatology, Amsterdam Rheumatology and Immunology Centre (ARC), Amsterdam UMC, Location AMC, University of Amsterdam, Amsterdam, Netherlands; ^2^Department of Experimental Immunology, Amsterdam Infection & Immunity Institute (AIII), Amsterdam UMC, Location AMC, University of Amsterdam, Amsterdam, Netherlands; ^3^Université Paris-Saclay, CEA, INRAE, Département Médicaments et Technologies pour la Santé, SIMoS, Gif-sur-Yvette, France; ^4^Department of Clinical Epidemiology, Biostatistics, and Bioinformatics, Amsterdam Infection & Immunity Institute (AIII), Amsterdam Public Health Research Institute, Amsterdam UMC, Location AMC, University of Amsterdam, Amsterdam, Netherlands

**Keywords:** T-cell receptor, adaptive immune receptor repertoire, T cell responses, next generation sequencing, bioinformatics, immunoinformatics

## Abstract

High-throughput T-cell receptor repertoire sequencing constitutes a powerful tool to study T cell responses at the clonal level. However, it does not give information on the functional phenotype of the responding clones and lacks a statistical framework for quantitative evaluation. To overcome this, we combined datasets from different experiments, all starting from the same blood samples. We used a novel, sensitive, UMI-based protocol to perform repertoire analysis on experimental replicates. Applying established bioinformatic routines for transcriptomic expression analysis we explored the dynamics of antigen-induced clonal expansion after *in vitro* stimulation, identified antigen-responsive clones, and confirmed their activation status using the expression of activation markers upon antigen re-challenge. We demonstrate that the addition of IL-4 after antigen stimulation drives the expansion of T cell clones encoding unique receptor sequences. We show that our approach represents a scalable, high-throughput immunological tool, which can be used to identify and characterize antigen-responsive T cells at clonal level.

## Introduction

In recent years, advances in high-throughput adaptive immune receptor repertoire sequencing (AIRR sequencing) made it possible to screen thousands and thousands of T-cell receptor (TCR) sequences at once. This constituted a major advance in the characterization of T cell responses in both health and disease [reviewed in (1)]. However, a major limitation of this approach is that it is not possible to derive information on the antigen specificity or functional phenotype of the receptor-carrying T cell from the TCR sequence itself.

In 2017 two computational approaches described the link between TCR sequences and antigen specificity in more detail (2, 3). However, these technologies require pre-existing knowledge and access to antigenic MHC–peptide complexes to identify the sequence patterns that can be used to predict the antigen-binding specificity of TCRs. In this area, novel approaches that identify TCR antigen-specificity groups in high-throughput without the need to isolate antigen-specific T-cells would be highly valuable (4). Still, the functional phenotype of the cell carrying the TCR would remain unknown.

The recent development of single-cell sequencing technologies opens the possibility to integrate the full T cell transcriptome to its receptor clonality. However, retrieving the TCR sequences from the transcriptome data has proven to be quite challenging and new bioinformatics methods had to be developed *ad hoc* (5–8). Very recently, the addition of a TCR-specific amplification and enrichment step made it possible to overcome this issue and obtain transcriptome and receptor sequences simultaneously (9). Despite the high resolution offered by this approach, the high cost associated together with the generally low frequencies of antigen-specific T cells in the repertoire prohibits high-throughput studies. As a result, studies that up to now have analyzed the receptor characteristics of antigen-specific T cells performed TCR repertoire sequencing on an already isolated population of cells (10–13). This includes cells sorted directly *ex-vivo* using MHC-antigen tetramers or cells stimulated *in vitro* with the given antigen and then sorted based on the expression of activation markers or their proliferative capacity. If MHC-antigen tetramers are nowadays considered as the golden standard for the isolation of antigen-specific T cells, they are quite difficult to apply in large-scale studies, especially when analysis concerns different antigen specificities in different MHC backgrounds. In addition, the ability of a T cell to bind a given antigenic protein/peptide through its T-cell receptor, *i.e.* its *specificity*, not always coincides with the ability of a T cell to get activated and undergo cellular proliferation and differentiation in response to the antigenic stimulus, *i.e.* its *responsiveness*. Anergic, senescent or exhausted T cells are an example of this. Furthermore, when analyzing antigen-induced responses in patients’ samples, the *in vitro* proliferation and activation responses detected may themselves not be specific for the antigen in question and may include bystander responses (14). We believe such responses, in spite of being not antigen-specific, may still have a substantial impact on the overall outcome of antigen-induced immune responses *in vivo*.

In this study we combine *in vitro* T cell stimulation assays, high-resolution UMI-based TCR repertoire sequencing of replicated samples and Fluorescence Activated Cell Sorting (FACS) in a high-throughput approach to obtain integrated information about antigen responsiveness, functional phenotype, and receptor clonality of individual T cell clones, with focus on CD4+ T cell responses. The application of two widely used RNA-sequencing algorithms, i.e. *K-means clustering* and *edgeR*, allowed us to explore the dynamics of clonal expansion after antigen stimulation and identify significantly expanded antigen-responsive clones. Antigen responsiveness was then confirmed by expression of the CD4-specific activation markers CD154 and CD25 upon antigen re-stimulation. Finally, we studied differentially expanding TCR clones in different cytokine milieus. We demonstrated that the presence of IL-4 after antigen stimulation is required to induce the expansion of clones with unique TCR sequences and the production of Th2-specific cytokines. We propose that this approach represents a scalable, and high-throughput innovative immunological tool that helps in linking antigen-responsiveness and functional phenotype to T-cell receptor clonality.

## Materials and Methods

### Samples and Peptides

Blood samples were obtained from healthy donors at Etablissement Français du Sang (EFS), Rungis, France, after informed consent from the donor and in accordance with EFS guidelines approved by the Comité d’éthique et de déontologie de l’EFS. Peripheral blood mononuclear cells (PBMCs) were isolated from total blood using Ficoll/Lymphoprep separation (GE Healthcare) and cryopreserved in liquid nitrogen until further use. Peptides used for the T cell stimulation assay were selected from published CD4+ T cell epitopes from Cytomegalovirus, Epstein–Barr virus and Influenza A virus and synthesized by Pepscan (The Netherlands). Peptides sequences are reported in **Supplementary Table S1**.

### Peptide-Based T Cell Stimulation Assay and FluoroSpot Assay

To investigate memory T cell responses at clonal level we adopted a short-term peptide-based T cell stimulation assay previously applied to study immunogenicity of compounds in immunized individuals (15). Briefly, frozen PBMCs derived from healthy donors were thawed and seeded at 5 × 10^6^ cells/ml in a 24-well plate in the presence of the pool of peptides (10 µg/ml/peptide) in RPMI 1640 medium supplemented with 5% human AB serum (Sigma-Aldrich), 60 U/ml IL-2 (R&D), 100 U/ml IL-4 (R&D) and 1 µg/μl of anti-CD28 (clone 15E8, Miltenyi Biotech). After two days of culture, the medium was changed to RPMI 1640 supplemented with 10% human AB serum and cytokines and refreshed every 2–3 days. At day 10, cells were harvested and incubated with medium, the pool of peptides (10 µg/µl/peptide) or phytohemagglutinin (PHA, 20 µg/ml) in AIM-V medium supplemented with 1.7 U/ml IL-7 (R&D) in Fluorspot pre-coated plates (Mabtech) at a concentration of 10^5^ cells/well. After 48 h cells were harvested, snap frozen in liquid nitrogen and preserved at −80°C until further analysis. INF*γ* and IL-5 spots were revealed according to the manufacturer’s instruction and counted with a computer-assisted video image analyzer (AID iSpot reader, AID, Strassberg, Germany). To test the impact of different cytokine environments, the same protocol described above was adopted, excluding both (“none” condition) or one (“IL-2” condition) of the added cytokines.

For the sorting of CD154+ CD25+ T cells, day 10 post-stimulation cultures were re-stimulated for 6 h as described above with the addition of 1 μg/ml of CD40 pure (Miltenyi Biotech). Cells were then harvested and stained with the following antibodies: anti-CD3-PE (clone UCHT1, Beckson Dickinson), anti-CD4-APC-H7 (clone RPA-T4, Beckson Dickinson), anti-CD154-APC (clone TRAP1, Beckson Dickinson), anti-CD25-PE-Cy7 (clone M-A251, Beckson Dickinson). Sorting was performed on a FACS ARIA III cytometer (Becton Dickinson, San Jose, CA, USA). For donor 2, after the 6 h re-stimulation in the presence of CD40, cells were harvested and enriched for CD154 expression using magnetic beads following the manufacturer’s protocol (CD154 MicroBead Kit from Miltenyi Biotec).

### Next-Generation Sequencing of the T-Cell Receptor Repertoire

**Figure 1:**
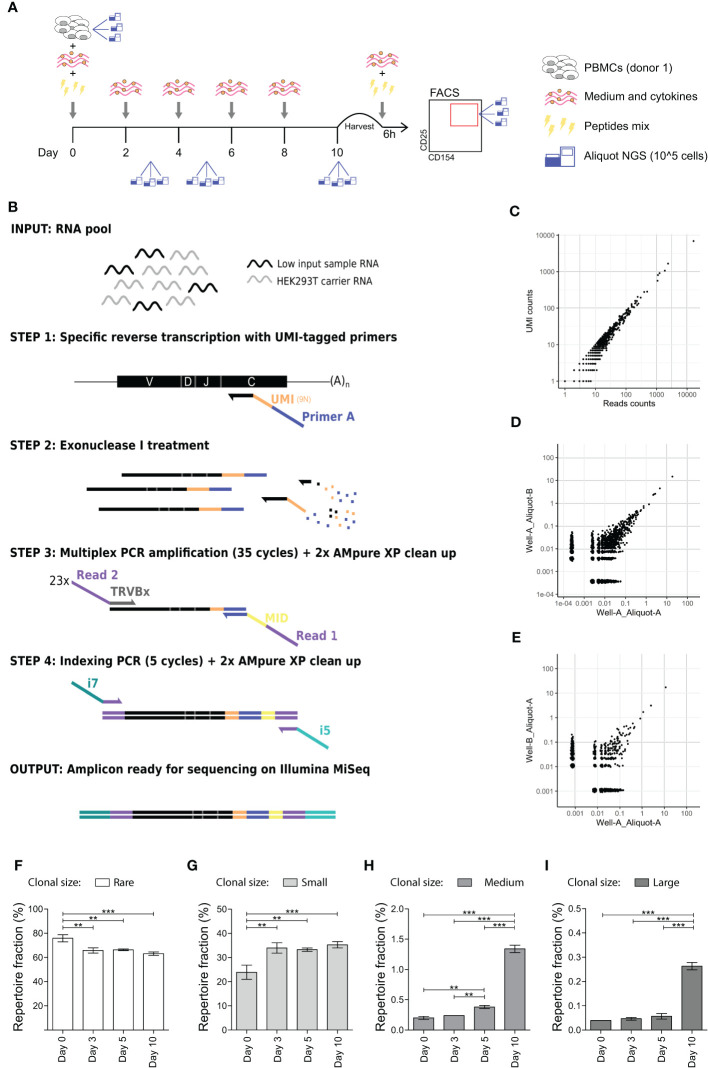
*In vitro* expansion to medium and large sized TCR clones occurs 5 and 10 days after antigen stimulation. **(A)** Schematic representation of the *in vitro* culture system. PBMCs are stimulated with a mix of peptides and kept in culture for 10 days. Medium and cytokines are refreshed every two days. At day 10 PBMCs are briefly re-stimulated with the peptides mix and then sorted on CD154 and CD25 expression. Aliquots for TCR analysis are taken at day 0 (before stimulation), day 3, day 5, and day 10 post-stimulation and after sorting. **(B)** Schematic representation of the high-throughput UMI-based TCR repertoire sequencing workflow adapted for low cells input. Sample’s RNA is mixed with carrier RNA from HEK293 cell (not expressing TCR) prior cDNA synthesis. Specific reverse transcription is performed with UMI-tagged primers complementary to the TRC*ß* constant chain (step 1). Exonuclease I treatment is performed to remove unbound reverse transcription primers (step 2) before performing a multiplexed PCR with 23 TCRß variable (TRBV) chain forward primers and a single reverse primer (step 3). Obtained amplicons are then indexed with i7 and i5 Nextera Illumina indexes (step 4) and sequenced on an Illumina MiSeq platform. **(C)** Scatter plot showing the overlap in the clonal frequency of individual TCR clones (black dots) when calculated on reads counts (x-axes) or UMI-counts (y-axes). **(D, E)** TCR repertoire overlap of technical replicates, *i.e.*, two different aliquots obtained from the same culture well **(D)** and of experimental replicates, *i.e.*, two different aliquots obtained from two different wells of the same culture **(E)**. Each dot represents an individual TCR clone, and its frequency in the two repertoires is depicted on the x- and y-axes as a percentage of total unique UMIs. **(F–I)** Clonal size distribution in the TCR repertoire at different time points during the *in vitro* culture from a single healthy donor. Each histogram depicts the percentage of the repertoire occupied by **(F)** rare (single UMI-count clones, clonal frequency (c.f.) = 0.004%), **(G)** small (0.004% < c.f. < 0.1%), **(H)** medium (0.1% ≤ c.f. <0.5%) or **(I)** large (c.f. ≥ 0.5%) TCR clones. Bars show mean and error bars show standard deviation (**p ≤ 0.01, ***p ≤ 0.001 using one-way ANOVA with Bonferroni correction for multiple testing).

As input material for next-generation sequencing of the T-cell receptor (TCR) repertoire we used snap frozen 10^5^ cell pellets collected in triplicate during the peptide-based T cell stimulation assay. Cells were lysed by direct addition of buffer RLT (Qiagen) containing 1% *ß*-mercaptoethanol (Sigma-Aldrich) to the snap frozen cell pellet, followed by vortexing. RNA extraction was performed using the RNeasy Micro Kit (Qiagen) according to the manufacturer’s instructions. For the amplification of TCR molecules we adopted a previously described protocol that we optimized for low cell input (**Figure 1B**). Instead of a total cDNA synthesis, we performed a specific cDNA synthesis of TCR*β* molecules using custom primers containing (from the 3′- to 5′-end): a specific sequence binding to the TCR *β*-chain constant region (16), a nine random nucleotides Unique Molecular Identifier (UMI) and a consensus sequence for the subsequent PCR amplification. In case of low target RNA input, RNA from the non-TCR expressing cell line HEK293T was added to the reaction to increase efficiency of the reverse transcription, which was performed on a total of 250 ng input RNA using SuperScript III Reverse Transcriptase (Thermo Fisher Scientific) according to the manufacturer’s instruction. To remove unbound cDNA synthesis primers, we added Exonuclease I (Thermo Fisher Scientific). Half of the cDNA synthesis volume was then used as input for a 35-cycles multiplexed PCR (17) using 23 forward primers covering all TCR beta chain variable genes (16) and a consensus reverse primer that contains an eight nucleotide-long molecular identifier (MID) for sample identification. Both forward and reverse PCR primers were tagged at the 5′ end with Read2 and Read1 sequencing primers from the MiSeq Nextera system (Illumina), respectively. The obtained PCR products were purified twice using AMPure XP beads (Beckman Coulter) in a 1:1 ratio and quantified using the Qubit dsDNA HS Assay Kit (Thermo Fisher Scientific). 50 ng of the purified PCR product was used as input for the indexing PCR which uses the Nextera i7 and i5 Index primers (Illumina). The indexed amplicons were again purified, quantified and sequenced using the Illumina Miseq Kit v3 2 × 300 bp technology according to the manufacturer’s manual (Illumina, San Diego, California, USA). All primers used in this protocol were ordered from Biolegio, Nijmegen, The Netherlands.

### T-Cell Receptor Repertoire Analysis

Sequencing reads were analyzed using an in-house developed workflow (RESEDA) for T- and B-cell repertoire analysis (https://bitbucket.org/barbera/reseda). Main steps in the RESEDA workflows are: assembly of paired-end reads using PEAR (18), MID-based demultiplexing of samples, alignment of the assembled reads with an in-house copy of VDJ genes from the IMGT database (19) using BWA MEM (20), V and J gene assignment and identification of the CDR3 (CDR3, Complementary Determining Region 3; V, Variable gene; J, Joining gene), removal of reads with low quality bases (Phred-score < 30) in the CDR3 region, clustering of reads in TCR clones based on 100% amino acid CDR3 identity, and UMI-based determination of the clonal frequency. Analysis of the clonal size distribution was performed using R (version 3.3.2) on downsized repertoires (downsized repertoire size used in **Figure 1**: 25,000 UMIs; downsized repertoire size used in **Supplementary Figure S5**: 5,000 UMIs). Analysis of the different profiles of clonal expansion of individual TCR clones was performed using k-means clustering. The R routine used for k-means clustering was the *k-means* from the *stats* package, which contains the implementation of the algorithms proposed by Macqueen (21), Hartigan and Wong (22). Only TCR clones present in at least two of the triplicate samples taken at each experimental time point were considered for the analysis. At each experimental time point UMI-corrected counts of TCR clones in the repertoires were averaged among triplicate samples and subsequently autoscaled (Z-score normalization). The changes in the autoscaled frequency between the pre-stimulation repertoire and the given post-stimulation timepoint repertoire were the information used to base the clustering upon. The optimal number of clusters was determined using the elbow method, based on the sum of squared errors (*i.e.*, the variance in the dataset) as a function of the clusters’ number (**Figure S1A** in **Supplementary Material**). We chose to allow 10 clusters as no substantial increase in the sum of squares error within each cluster was observed when allowing more clusters indicating that the majority of the dataset variance is explained within 10 clusters. Since k-means clustering is sensitive to the initial choice of centroids we reinitiated the procedure 25 times.

For the identification of differentially expanded clones, we applied *edgeR*, a widely used approach in RNA-seq gene expression profiling that estimates differential gene expression based on a negative binomial distribution, taking into account biological replicates (23, 24). Non-normalized UMI-corrected counts of TCR clones in repertoires obtained in triplicate at the different experimental time points were used as input. Cut-offs for differential expansion were set at a fold-change above 1.5, or below −1.5, and an adjusted p-value below 0.05 after Benjamini–Hochberg correction for multiple comparison. Raw sequencing reads have been deposited at NCBI Sequence Read Archive (BioProject: PRJNA685965) and processed repertoires are available upon request to the corresponding author.

### Statistics

Data are presented as mean and standard deviation (SD) after performing the D’Agostino and Pearson omnibus test for normality. Differences between groups were evaluated using one- or two-way analysis of variance (ANOVA) followed by Bonferroni’s multiple comparison post-test; p-values ≤0.05 were considered statistically significant. Prism 7 software (Graph Pad, San Diego, CA, USA) was used to perform the statistical tests.

## Results

### *In Vitro* Expansion to Medium and Large Sized TCR Clones Occurs 5 and 10 Days After Antigen Stimulation

To study memory antigen-responsive CD4+ T cells in an *in vitro* model system, we stimulated PBMCs from a single healthy donor with a peptides’ mix of published CD4+ T cell epitopes derived from Cytomegalovirus, Epstein–Barr Virus, and Influenza A virus to benefit from the natural immunity against these common viruses (**Table S1** in **Supplementary Material**) (25–27). After 2 days of stimulation, medium was changed and cultures were maintained in the presence of cytokines for another 8 days. We collected cellular samples in triplicate prior to stimulation (day 0) and at different time points after stimulation (day 3, 5 and 10) (**Figure 1A**). Quantitative amplification of the TCR repertoire was performed using a previously validated protocol (17), optimized for low cell inputs and modified to incorporate unique molecular identifiers (UMI) to correct for potential amplification bias (**Figure 1B**). We evaluated the protocol regarding estimation of clonal frequencies (**Figure 1C**), and technical and experimental reproducibility (**Figures 1D, E**).

Subsequently, we investigated whether we could detect clonal expansion after *in vitro* stimulation in the TCR repertoire by analyzing the frequency distribution of clones in the repertoire, *i.e.*, the clonal size distribution. The fraction of the repertoire occupied by rare clones (*i.e.*, clones detected only once, clonal frequency (c.f.) = 0.004% in the downsized dataset) significantly decreased at day 3 (p = 0.001) (**Figure 1F**). Conversely, we observed a significant increase in the fraction of small clones (0.004% < c.f. < 0.1%) from day 3 onwards (**Figure 1G**), of medium-sized clones (0.1% ≤ c.f. <0.5%) from day 5 onwards (**Figure 1H**) and of large clones (c.f. ≥ 0.5%)) at day 10 (**Figure 1I**). The overall change from day 0 to day 10 in the fraction of repertoire occupied was 75.8 ± 2.97 to 63.1 ± 1.39 for rare clones (mean ± SD; p-value = 0.001), 23.9 ± 2.95 to 35.3 ± 1.32 for small clones (p = 0.002), 0.20 ± 0.03 to 1.34 ± 0.06 for medium-sized clones (p ≤ 0.001), and 0.04 ± 0.00 to 0.26 ± 0.01 for large clones (p ≤ 0.001). In conclusion, clonal expansion to medium and large sized clones can be detected in the repertoire obtained from *in vitro* cultures after 5 days of antigen stimulation, while no such changes occurred in the first 3 days after antigenic challenge.

### Only Few TCR Clones Show Increased Clonal Frequency After 10 Days of *In Vitro* Antigen Stimulation

To get more insight into the clonal expansion patterns underlying the clonal size increases observed in **Figures 1F–I**, we sought to identify and differentiate the different expansion profiles of single TCR clones in response to antigen stimulation using existing bioinformatic routines. The final aim was to detect, in a later analysis, which expansion profiles best matched those of antigen-responding clones. To this end, we implemented the k-means clustering, a validated statistical approach widely used in gene expression analysis (28). When applied to our repertoire data (from **Figure 1**), the k-means algorithm grouped together TCR clones with similar expansion profiles during the 10-days culture into ten clusters (**Figure S1B** in **Supplementary Material**). The results of this unsupervised clustering indicated clusters 1 and 4 as the most abundant clusters containing 28.1 and 27.5% of the TCR clones, respectively (**Figure S1B** in **Supplementary Material**). The clones in these clusters showed a transient increase in clonal frequency respectively at day 3 and day 5 post-stimulation. Also clones in cluster 8 (1.85%) showed a transient peak covering day 3 and 5 post-stimulation. Clones in cluster 5, the third largest cluster (22.9%), showed an immediate decrease in clonal frequency at day 3 persisting up to day 10. The additional clusters in which the clonal frequency decreased from day 0 to day 10 were clusters 6 (1.45%), 7 (1.46%) and 10 (1.65%).

The remaining three clusters included clones with an increased frequency at the end of the culture. Clones in cluster 3 (12.4%) showed an increase in frequency from day 5 to day 10 only, while clones in cluster 2 (1.6%) showed an increase in frequency already at day 3 up to day 10. Clones in cluster 9 (0.97%) showed a transient increase from day 0 to day 3 followed by a second increase from day 5 to day 10. The impact (*i.e.*, the percentage of total repertoire occupied) of the clones allocated to clusters 2, 3 and 9 was 0.76, 1.71, and 0.34% on the day 0 repertoire, and 40.8, 34.4, and 2.17% on the day 10 repertoire, respectively.

Taken together, these results reveal that only 15% of all the clones cultured show an increase in frequency during the 10 days stimulation assay, with a collective impact on the total repertoire that rises from 2.81% before stimulation to 77.31% after stimulation.

### Identification of Antigen-Responsive Expanding TCR Clones

**Figure 2:**
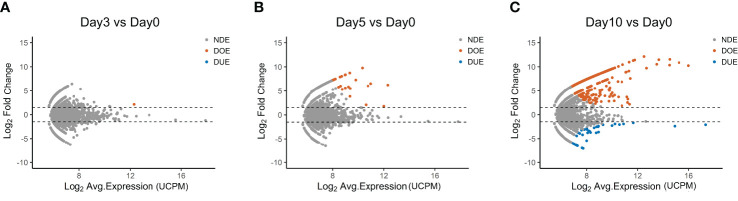
Selection of Differentially Expanding TCR clones using *edgeR*. **(A–C)** MA plots showing the differential expansion of individual TCR clones calculated with *edgeR* between the day 0 and **(A)** day 3, **(B)** day 5, or **(C)** day 10 repertoire. Each dot represents an individual TCR clone where the x- and y-axes depict the average log_2_ clonal frequency (calculated from UMI counts per million; UCPM) and log_2_ fold change compared to day 0 respectively. Differentially (Benjamin–Hochberg corrected p-value <0.05) Over-Expanded clones (DOE; fold changes >1.5) are colored in orange, Differentially Under-Expanded clones (DUE; fold changes < −1.5) are colored in blue, while Not Differentially Expanded clones (NDE, −1.5 < fold changes < 1.5) are colored in gray.

The data presented up to now show that only few TCR clones in the repertoire do expand after 10 days of antigen-stimulation, indicating antigen responsiveness. To identify these antigen-responsive clones we applied another algorithm developed for the analysis of RNA-seq data, namely *edgeR* (**Figure 2**)*. EdgeR* is used to perform differential expression analysis on gene expression data to identify over- and under-expressed genes (23, 24). Applied to our TCR repertoire data (from **Figure 1**), *edgeR* allows the identification of over- and under-*expanded* TCR clones, *i.e.*, clones with respectively an increased or decreased clonal frequency in the post-stimulation repertoire. We hereafter name such clones as “Differentially Over-Expanded (DOE)” and “Differentially Under-Expanded (DUE)”. When comparing the day 0 to the day 3 post-stimulation repertoire, only one clone was selected as Differentially Over-Expanded (DOE) (**Figure 2A**). The number of DOE-TCR clones increased to 25 at day 5 and to 548 at day 10 post-stimulation (**Figures 2B, C**). At day 3 and 5, none of the clones was selected as DUE. However, at day 10, 45 TCR clones were found to be DUE, some still showing a high clonal frequency in the total day 10 repertoires (**Figure S2** in **Supplementary Material**).

In **Table S2** in **Supplementary Material** the DOE clones are allocated to the different k-means clusters presented previously. As expected, the 548 DOE-TCR clones identified at day 10 were part of clusters 3 (n = 388), 2 (n = 134), and 9 (n = 24; two of the DOE-TCR clones were excluded from the clustering analysis because they were present in only one of the triplicates).

We conclude that differential expression analysis in TCR repertoires from triplicate samples can be used to identify consistently expanding TCR clones (DOE clones) during a 10 days culture.

### Expression of the Activation Markers CD154 and CD25 Confirms Antigen-Responsiveness of Differentially Over-Expanded TCR Clones

**Figure 3:**
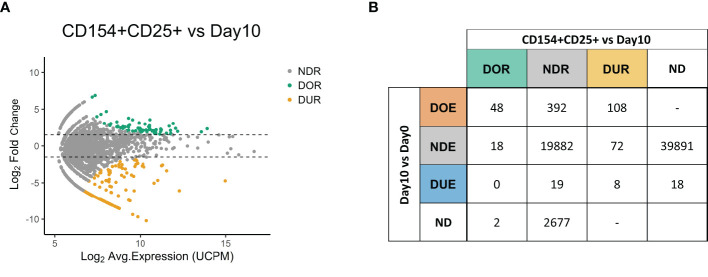
Expression of the activation markers CD154 and CD25 confirms the antigen-responsiveness of Differentially Over-Expanded TCR clones. **(A)** MA plot showing the changes in frequency in individual TCR clones when comparing the CD154+CD25+ sorted fraction after re-stimulation with antigen at day 10 to the day 10 repertoire. Each dot represents an individual TCR clone where the x- and y-axes depict the average log_2_ clonal frequency (calculated from UMI counts per million; UCPM) and log_2_ fold change compared to day 10 respectively. Differentially (Benjamini–Hochberg corrected p-value <0.05) Over-Represented clones (DOR, fold changes >1.5) are colored in green, Differentially Under-Represented clones (DUR, fold changes < −1.5) are colored in yellow, while Not Differentially Represented clones (NDR, −1.5 < fold changes <1.5) are colored in gray. **(B)** Cross-table showing the overlap between clones defined as Differentially Over-Expanded (DOE), Not Differentially Expanded (NDE) or Differentially Under-Expanded (DUE) in the day 10 to day 0 repertoire comparison, and clones defined as Differentially Over-Represented (DOR), Not Differentially Represented (NDR) or Differentially Under-represented (DUR) clones in the sorted CD154+CD25+ fraction to day 10 repertoire comparison. ND, Not Detected.

After the identification of differentially expanded TCR clones we aimed to validate the antigen responsiveness of the selected hits (**Figure 3**). At the end of the 10-day culture previously described (**Figure 1A**), T cells were briefly re-challenged with the pool of virus-derived peptides, and then FACS-sorted in triplicate based on the expression of CD4 and the CD4-specific activation markers CD154 and CD25 (**Figure 1A** and **Figure S3** in **Supplementary Material**). We sequenced the TCR repertoire of the sorted samples and compared these repertoires to the repertoires obtained prior to sorting at day 10. Using *edgeR* we identified 68 TCR clones with a significantly increased frequency in the sorted fraction compared to the pre-sorting day 10 repertoire, indicating an enrichment for the expression of the activation markers CD154 and CD25 (**Figure 3A**). These clones are hereafter referred to as “Differentially Over-*Represented* (DOR)” to distinguish them from the “Differentially Over-*Expanded* (DOE)” clones previously identified when comparing pre- and post-stimulation repertoires. In addition, we identified 188 “Differentially Under-*Represented* (DUR)” clones, *i.e.*, clones with a significantly lower frequency in the sorted fraction.

We subsequently investigated the relation between Differential Over-Representation in the CD154+ CD25+ sorted fraction with Differential Over-Expansion in the 10-days culture (**Figure 3B**). Of the 68 TCR clones that were Differentially Over-*Represented* (DO*R*) after the sorting, 48 (71%) were already identified as DO*E* in the 10-day culture, 18 (26%) as Non-Differentially *Expanded* (ND*E*), and none as DU*E*. Thus, the major fraction of the DOR clones (71%) can be captured as DOE clones in a simple 10-days culture.

Looking in the reverse direction, out of the 548 Differentially Over-*Expanded* (DO*E*) TCR clones identified in the 10 days culture, 474 were retrieved in the CD154+CD25+ sorted fraction (**Table S3** in **Supplementary Material**). If we assume equal TCR expression among the cells, the DOE-TCRs accounted for 61.4% of the cells in the day 10 sample, while they constituted 86.9% of the cells after sorting. Of the 548 clones selected as DO*E* in the 10-days culture, 108 (20%) were assigned to the DU*R* group, indicating that these clones did lack expression of CD154, CD25 or both upon re-stimulation with antigen. The remaining DOE clones were identified as DO*R* clones (N = 48 (8.8%)) or as Non-Differentially Represented clones (NDR; N= 392 (72%)), indicating a high or average expression of both CD154 and CD25. These results were reproduced in an additionally tested healthy donor (**Figure S4** in **Supplementary Material**).

To conclude, DOE clones were overrepresented in the TCR repertoire after sorting on CD154 and CD25 following brief antigen re-challenge, thus confirming antigen responsiveness. These DOE clones account for 71% of the clones Differentially Over-Represented after sorting, and are estimated to account for 87% of the cells after sorting.

### Antigen Stimulation in Different Cytokine Milieus Activates Different TCR Clones That Show Different Profiles of Cytokine Secretion

**Figure 4:**
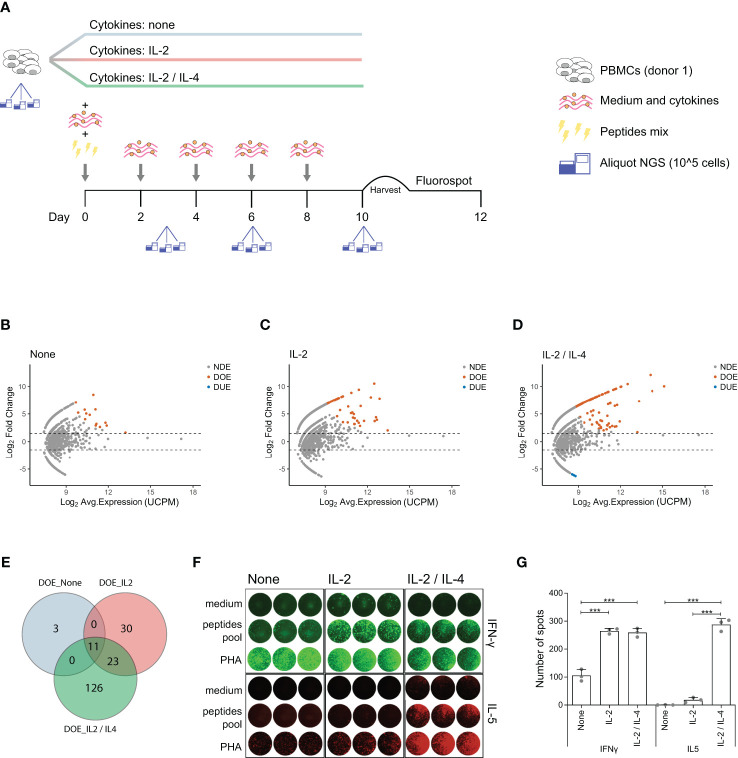
IL-4 induces expansion of antigen-responsive, IL-5 producing TCR clones with unique TCRs. **(A)** Schematic representation of the *in vitro* culture system modified to include different T cell skewing cytokines. PBMCs are equally divided in three fractions and then cultured separately in three different cytokines milieus: no cytokines, only IL-2 or IL-2 and IL-4 combined. All the other culture conditions are the same as described in **Figure 1**. At day 10 PBMCs are harvested and plated for the FluoroSpot assay. Aliquots for TCR analysis are taken at day 0 (before stimulation), day 3, day 6, and day 10 post-stimulation. **(B–D)** MA plots showing the differential expansion of individual TCR clones between the day 0 and day 10 repertoire in *in vitro* cultures after antigen stimulation in presence of **(B)** no cytokines, **(C)** IL-2 only or **(D)** both IL-2 and IL-4. Each dot represents an individual TCR clone where the x- and y-axes depict the average log_2_ clonal frequency (calculated from UMI counts per million; UCPM) and log_2_ fold change compared to day 0 respectively. Differentially (Benjamini–Hochberg corrected p-value <0.05) Over-Expanded clones (DOE; fold changes > 1.5) are colored in orange, Differentially Under-Expanded clones (DUE; fold changes < −1.5) are colored in blue, while Not Differentially Expanded clones (NDE, −1.5< fold changes < 1.5) are colored in gray. **(E)** Venn diagram showing the overlap of clones selected as Differentially Over-Expanded (DOE) in the day 10 repertoire of *in vitro* stimulated cultures in presence of no cytokines (light blue circle), IL-2 only (rose circle) or IL-2 and IL-4 (green circle). **(F)** Results of the FluoroSpot assay for the detection of T cells that produce interferon-***γ*** (IFN-***γ***) (FITC filter, *top panel*) and interleukin-5 (IL-5) (Cy5 filter, *bottom panel*) re-stimulated after 10 days of growth in different cytokine milieus with PHA, a pool of antigenic peptides or medium. **(G)** Histograms depicting the mean (±SD) number of T cell spots producing IFN-***γ*** (left panel) or IL-5 (right panel) from the FluoroSpot assay performed at the end of the *in vitro* culture in the presence of no cytokine, IL-2 only or both IL-2 and IL-4 from a single healthy donor (***p ≤ 0.001 using one-way ANOVA followed by Bonferroni’s Multiple comparison post-test).

Differential expression analysis in TCR repertoires obtained before and after *in vitro* antigen stimulation allowed us to link antigen-responsiveness to clonal TCR sequences. To investigate whether these antigen-responsive TCR clones had a specific functional phenotype, we modified our *in vitro* model to include different CD4+ T cell skewing cytokines. A new culture was started and PBMCs from the same donor showed in **Figure 1** were stimulated using the same pool of virus-derived peptides but were incubated from the second day after stimulation in medium containing either no supplementary cytokine, or IL-2 alone, or IL-2 and IL-4 together. At day 0 (pre-stimulation) and at day 3, 6, and 10 post-stimulation we collected triplicate samples for TCR repertoire analysis (**Figure 4A**). Of note, at day 6 and day 10 the number of large clones (clonal frequency ≥ 0.5%) was significantly increased when both IL-2 and IL-4 were added to the culture medium, compared to conditions where only IL-2, or no cytokines were added (**Figure S5** in **Supplementary Material**). We identified DOE-TCR clones after 10 days of culture in the different cytokine milieus (**Figures 4B–D**). If no cytokines were present, or if only IL-2 was added, respectively 14 and 64 TCR clones were selected as DOE after 10 days of culture (**Figures 4B, C**). In the presence of both IL-2 and IL-4, we identified 160 DOE-TCR clones (**Figure 4D**). Interestingly, out of the 160 clones selected in presence of IL-2 and IL-4, only 34 (21%) were also identified in the other two conditions tested: 23 clones were also retrieved as DOE if only IL-2 was present during culture, while 11 were retrieved as DOE both when IL-2 or no cytokines were present during culture (**Figure 4E**). Thus, the addition of IL-4 after antigen stimulation induces the expansion of antigen-responsive TCR clones with unique receptor sequences different from those observed in absence of IL-4.

To analyze the functional characteristics of the IL-4 induced clones we measured the secretion of IFN-*γ* (Th1 specific cytokines) and IL-5 (Th2 specific cytokine) after re-stimulation with the pool of virus-derived peptides using a Fluorospot assay (**Figure 4F**). IFN-*γ* producing T cell spots were detected in a significantly higher number when IL-2 or when both IL-2 and IL-4 were added to the medium (mean ± SD: 265 ± 10 for IL-2, 259 ± 16 for IL-2 and IL-4 *versus* 105 ± 21 for no cytokines; both p < 0.001). In contrast, IL-5 producing T cell spots were clearly detected only in the presence of IL-2 and IL-4 (mean ± SD: 287 ± 21 for IL-2 and IL-4 *versus* 18 ± 9 for IL-2 and 0 ± 1 for no cytokines; both p < 0.001) (**Figure 4G**).

All together, these data show that addition of IL-4 after antigen stimulation induces expansion of unique antigen-specific TCR clones and production of Th2 specific cytokines.

## Discussion

In this study we combined *in vitro* T cell stimulation assay, AIRR sequencing and bioinformatics methods developed for RNA sequencing to identify and further characterize antigen-responsive TCR clones. This approach allowed us to obtain integrated information about antigen responsiveness, functional phenotype and receptor clonality of CD4+ T cells at the clonal level.

The observed dynamics of TCR clonal expansion after *in vitro* stimulation with the pool of viral peptides (**Figures 1F–I**) recapitulates previously published *in vivo* and *in vitro* observations on T cell responses to viral infection (29). Here, almost no cellular expansion of CD4+ T cells was observed in the initial four days post-infection. However, after day 4, a massive exponential growth of the viral specific CD4+ T cell population was observed, with a ~150-fold increase. In agreement with these data, we observed a significant increase in medium and large clones at day 5 and day 10 post-stimulation, while such increase was not observed at day 3. In addition, in the day 5 and the day 10 repertoire we detect TCR clones with a ~30 to ~1000-fold change in frequency compared to the day 0 repertoire (**Figures 2B, C**). Thus, the dynamics of clonal expansion observed in the *in vitro*-derived TCR repertoires recapitulate the dynamics of antigen-specific expansion observed in *in vivo* settings.

Selection of antigen-responsive TCR clones from immune receptor repertoire data was previously based on changes in the ranking of clones in repertoires obtained before and after antigen stimulation (30), differences between repertoires obtained from antigen-stimulated *versus* non-antigen-stimulated cultures (31), or repertoire analysis in an enriched fractions of antigen-responding T cells (32, 33). As shown in our data, the post-stimulation repertoire may still contain very dominant TCR clones that do now show a significant over-expansion after antigen-stimulation but rather almost maintain the same pre-stimulation frequency (**Figure S2** in **Supplementary Material**). Therefore, clonal dominance at a single time point is probably not very informative regarding antigen-responsiveness. We selected antigen-specific clones based on the statistically significant changes in clonal frequency between the pre- and post-stimulation repertoires while accounting for experimental variability using replicate samples. The approach taken adjusts for different sequencing depth and therefore can be directly applied to differently-sized TCR repertoires without a need for any repertoire equalization (or downsizing) that in most of the cases leads to substantial loss of information. Thus, this approach can be used as routine workflow for quantitative analysis in immune receptor repertoire, the same way it is routinely used for the analysis of transcriptome data.

We observed that clones selected as Differentially Over-Expanded (DOE) in the 10 days culture are more often selected also as Over-*Represented* (DO*R*) in the repertoire of sorted CD4+ T cells expressing the activation markers CD154 and CD25 after antigen re-challenge (**Figure 4B**). However, it should be noted that a large fraction of the identified DOE clones at day 10 is selected as Non-Differentially Represented (NDR) or even Under-Represented (DUR) after sorting. Since our sorting strategy required both CD154 and CD25 expression, we can’t exclude that some of the *over-expanded* clones detected at day 10 were actually single positive for any of the two activation markers tested and therefore still be antigen-responsive despite being selected as NDR or DUR. Likewise, only 48 of the 66 DOR clones were identified as DOE clones, suggesting that the remaining 27% of the over-represented clones after sorting did not undergo differential clonal expansion during the *in vitro* stimulation experiment. These results indicate that the optimal strategy to identify antigen-responsive T cells might require a combination of both approaches. In addition, further analysis might be needed to discriminate between specific and bystander T cell clonal responses against the given antigen. Even though we think that in clinical settings both responses do contribute to the overall antigen-induce immune response and are therefore both of interest, other experimental settings might require this distinction to be made.

When evaluating the *in vitro* T cell clonal responses in different cytokine milieus we observed two interesting groups of differentially expanding TCR clones. The first group of TCR clones was able to differentially over-expand when adding IL-2 only or when adding both IL-2 and IL-4 after the same antigen stimulation, suggesting that the same T-cell receptor is expressed by cells coming from different T helper subsets. This has been already shown for T cells responsive to bacterial and fungal antigens (34), indicating that the same naïve T cell might be able to differentiate into two distinct functional memory subsets. However, another bigger group of TCR clones expanded only when IL-4 was added to the system, indicating that the IL-4 driven clones carry unique TCRs. Since the production of IL-5 by Fluorospot was detectable only in presence of IL-4, this suggests that those clones were of the Th2 phenotype. Yet, further studies are needed to confirm this, *e.g.*, by showing that the IL-2 and IL-4 specific DOE-TCRs can be retrieved in the repertoires of IL-5 producing T cells. Another important question still open is whether these two T cell populations, in spite of having different TCRs and being of different phenotype actually respond to the same antigenic epitopes. It would be of interest to explore whether individual T cell epitopes elicit a phenotypically different T cell response and investigate the plasticity of such commitment or vice versa, whether different cytokines induce T cell responses with different epitope specificities. The use of a mix of peptides in our experimental settings hindered the investigation of these points for which the use of single-peptide T cell stimulation cultures should be applied. In such setting, TCR characteristics such has V- and J-gene usage and CDR3 sequence similarity could be further analyzed in order to identify specific patterns for epitope recognition.

Finally, we would like to emphasize an important advantage of this TCR repertoire-based approach. In fact, the receptor sequences of the identified antigen-responsive T cell clones can be used as a “fingerprint” to trace back these same T cell clonal responses in different samples derived from the same donor. For example, *in vitro* identified antigen-responsive TCRs can be traced back in *ex-vivo* derived samples taken at different time points, *e.g.*, in patients undergoing immunotherapy or vaccination, or in samples taken from different anatomical compartments. Such studies might be extremely helpful in further dissecting the temporal, spatial and functional evolution of antigen-responsive T cells in both physiological and pathological conditions. Furthermore, studies performed on a larger number of individuals would further extend the biological usefulness of this approach beyond the *in-vitro* conditions.

To conclude, we demonstrated that analysis of the differential expansion of individual TCR clones before and after *in vitro* antigen stimulation identifies antigen-responsive TCRs. In addition, we showed how further phenotypical and functional characterization of the selected TCR clones can be easily associated by applying different *in vitro* culture settings or performing additional cellular sorting. As such, this approach represents a statistically validated, scalable, innovative immunological tool that can be used to identify and characterize relevant antigen-driven T cell responses in both health and disease.

## Data Availability Statement

The datasets presented in this study can be found in online repositories. The names of the repository/repositories and accession number(s) can be found below: https://www.ncbi.nlm.nih.gov/, PRJNA685965.

## Ethics Statement

The studies involving human participants were reviewed and approved by Comité d’éthique et de déontologie de l’Etablissement Français du Sang (EFS), Rungis, France. The patients/participants provided their written informed consent to participate in this study.

## Author Contributions

NV, BM, SP, and MB contributed to design of the study. SP, MB, GB, and IN contributed to the acquisition and processing of samples. SP, MB, GB, AJ, BS, AK, BM, and NV contributed to data analysis and interpretation. All authors contributed to the article and approved the submitted version.

## Funding

The research leading to these results has received support from the Innovative Medicines Initiative Joint Undertaking under grant agreement n° 115303 (ABIRISK), resources of which are composed of financial contribution from the European Union’s Seventh Framework Programme (FP7/2007-2013) and EFPIA companies’ in kind contribution and was also supported by ZonMw, the Netherlands Organisation for Health Research and Development, in the program 2Treat (Grant 436001001).

## Conflict of Interest

The authors declare that the research was conducted in the absence of any commercial or financial relationships that could be construed as a potential conflict of interest.
