# Supplementary material for: Characterization and Monitoring of Antigen-Responsive T Cell Clones Using T Cell Receptor Gene Expression Analysis
Source: Front Immunol. 2021 Feb 19;11:609624. doi: 10.3389/fimmu.2020.609624 (PMC7932994; doi:10.3389/fimmu.2020.609624)
Supplement: Supplementary file 1 [file DataSheet_1.pdf]

Characterization and monitoring of antigen-responsive T cell clones using T cell receptor gene expression analysis

Sabrina Pollastro[1,2§], Marie de Bourayne[3§], Giulia Balzaretti[1,2§], Aldo Jongejan[4], Barbera D.C. van Schaik[4], Ilse T.G. Niewold[1], Antoine H.C. van Kampen[4], Bernard Maillère[3], Niek de Vries[1,2*].

[1]Department of Clinical Immunology & Rheumatology | Amsterdam Rheumatology and Immunology Centre (ARC), Amsterdam UMC, location AMC, University of Amsterdam, Amsterdam, The Netherlands.

[2]Department of Experimental Immunology | Amsterdam Infection & Immunity Institute (AIII), Amsterdam UMC, location AMC, University of Amsterdam, Amsterdam, The Netherlands.

[3]CEA-Saclay, Université Paris-Saclay, SIMOPRO, 91191 Gif-sur-Yvette, France.

[4]Department of Clinical Epidemiology, Biostatistics, and Bioinformatics, | Amsterdam Infection & Immunity Institute (AIII) | Amsterdam Public Health research institute, Amsterdam UMC, location AMC, University of Amsterdam, Amsterdam, The Netherlands.

§These authors equally contributed to this work

***Correspondence**:
Niek de Vries
n.devries1@amsterdamumc.nl

SUPPLEMENTARY MATERIAL

A

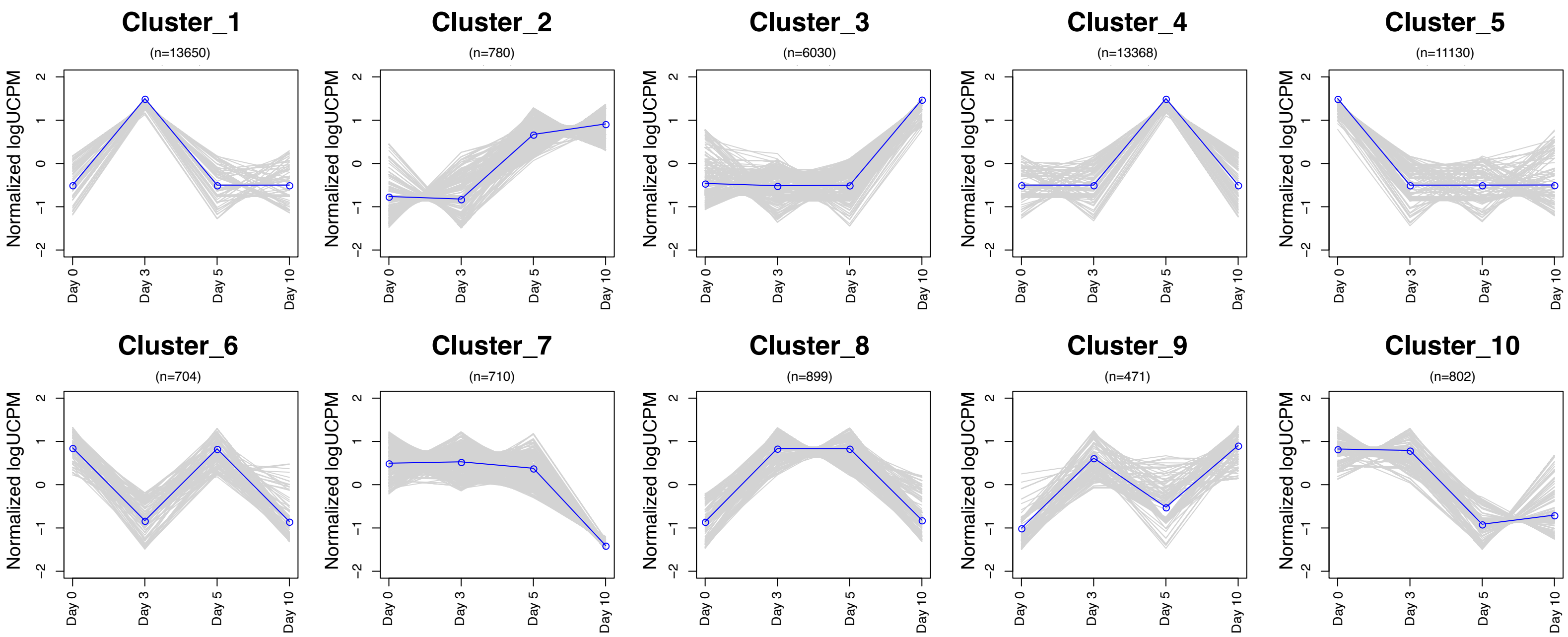

B

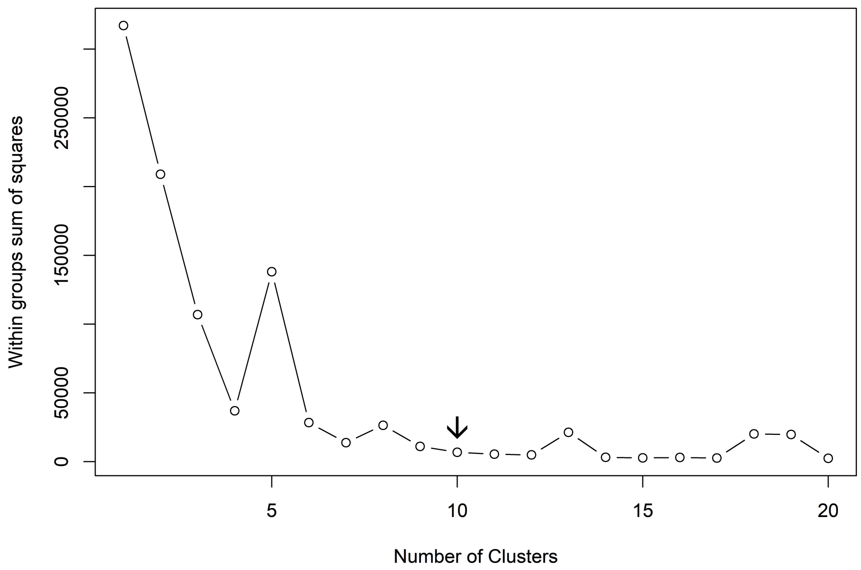

Figure S1: Analysis of different profiles of clonal expansion after in-vitro antigen stimulation in single TCR clones using K-means clustering. A) K-means clustering grouped TCR clones in 10 clusters based on the relative changes in clonal frequency at different time points after in-vitro stimulation. In each plot, the grey lines show changes in clonal frequency of individual TCR clones (depicted as normalized log2 UMI counts per million (UCPM); y-axes) at the different time points analyzed (x-axes), while the blue line represents the averaged profile of each cluster. B) Selection of the optimal number of clusters for K-means clustering using the elbow method. The scatter plot depicts the within sum of squares (y-axes) in relation to increasing number of allowed clusters (x-axes). The black arrows indicated the chosen number of clusters for the analysis reported in A.

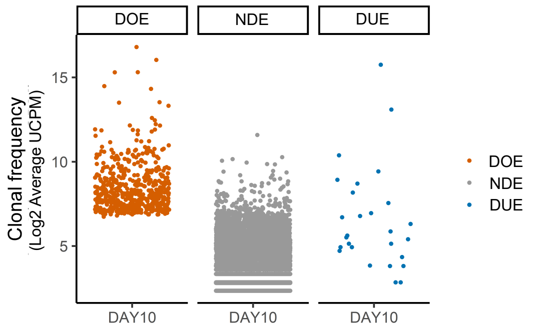

Figure S2: Clonal distribution of differentially expanded TCR clones in the day 10 post-stimulation repertoire. Scatter plots showing the frequency in the day 10 post-stimulation repertoire of TCR clones selected as Differentially Over-Expanded (DOE; first panel, orange dots), Not Differentially Expanded (NDE; second panel, grey dots) or Differentially Under-Expanded (DUE; third panel, blue dots) in the day 10 to day 0 repertoire comparison. Single dots represent unique TCR clones and y-axes depict the average log2 clonal frequency (calculated from UMI counts per million; UCPM) averaged among triplicates.

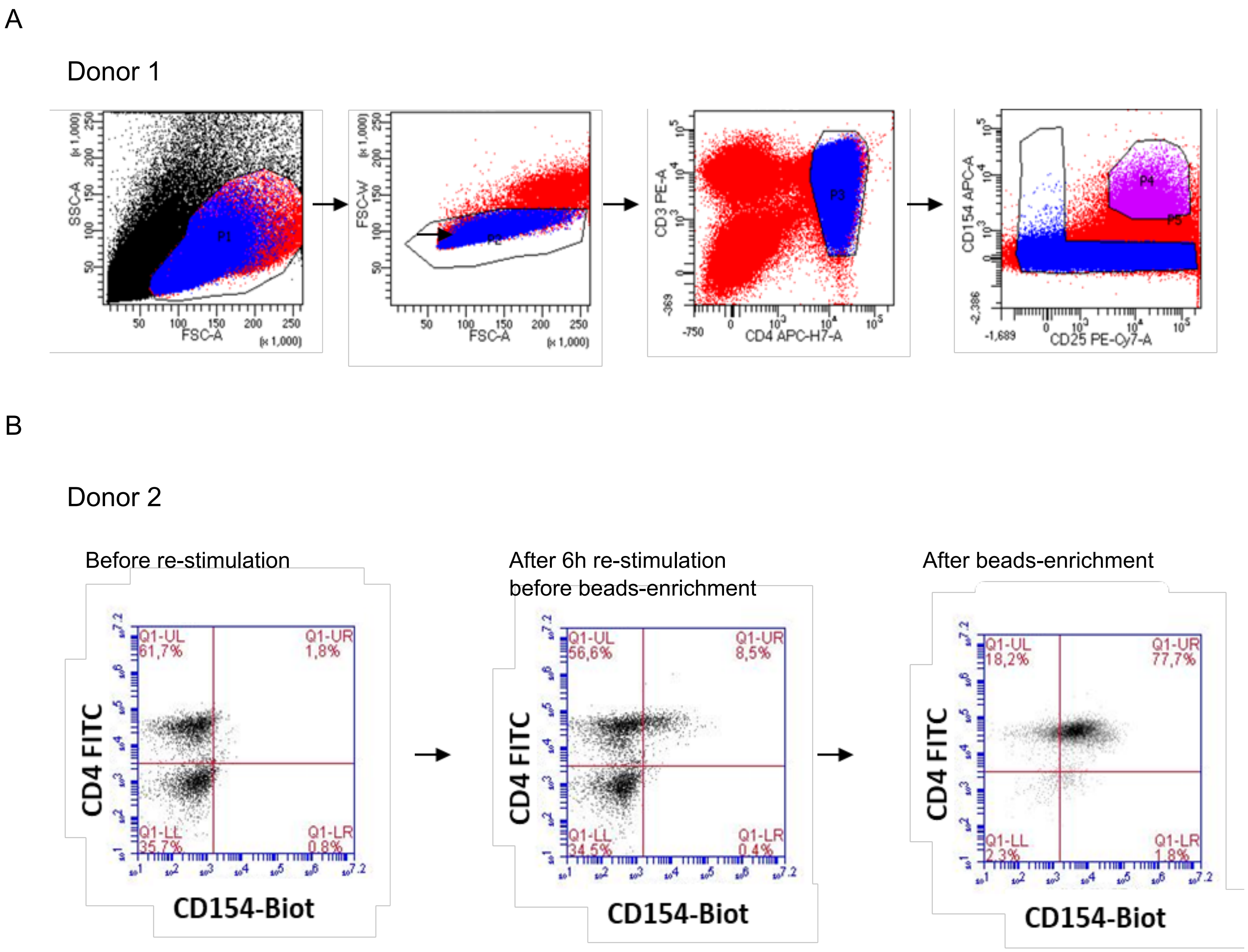

Figure S3: Strategies for the enrichment of activated CD4+ T cells at the end of the 10 days culture.
A) FACS plots showing the gating straptegy applied for donor 1 for the sorting of CD4+CD154+CD25+ cells (P4). B) FACS plots showing the percentage of CD4+CD154+ cells in donor 2 before the 6h antigen re-stimulation, after the 6h antigen re-stimulation but before CD154 beads enrichment and after CD154 beads enrichment.

A

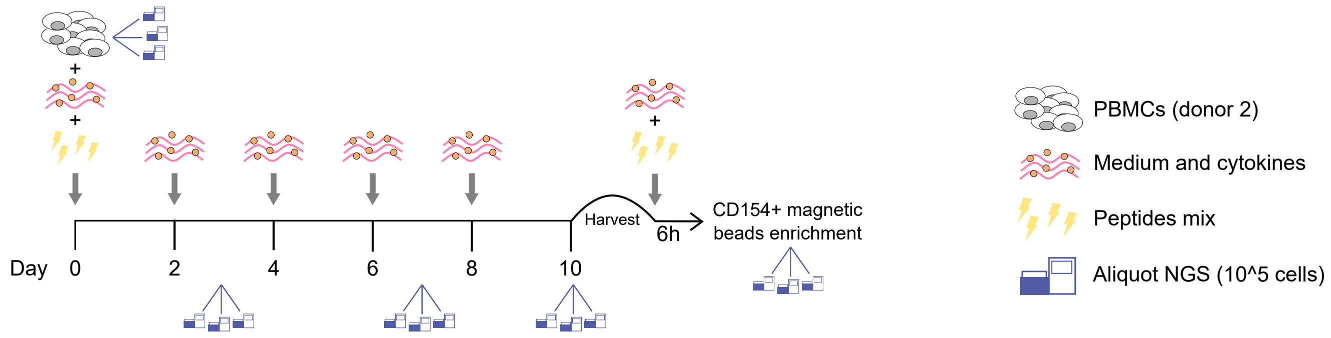

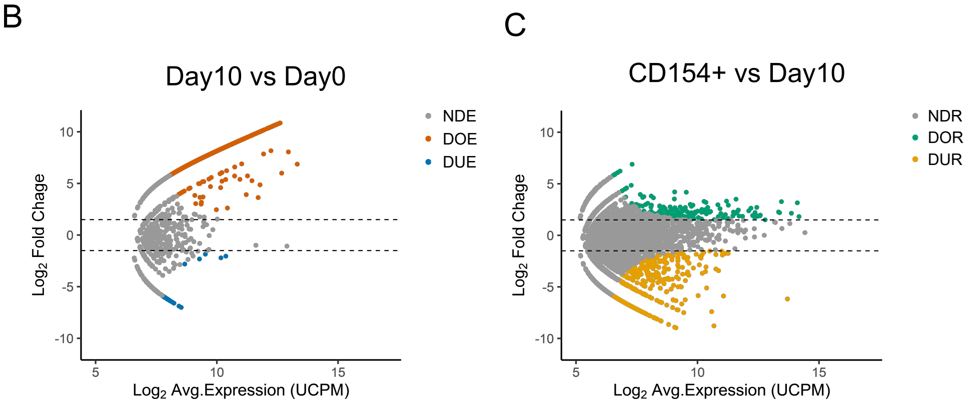

D

Day10 vs Day0 \ CD154+ vs Day10	DOR	NDR	DUR	ND
DOE	111	717	358	-
NDE	45	18351	54	4036
DUE	0	3	3	21
ND	7	4344	-	

DOE = 9% DOR, 61% NDR, 30% DUR
DOR = 70% DOE, 28% NDE, 0% DUE

E

Repertoire	Total clones in the repertoire	Number of DOE clones* in the repertoire	Impact of DOE clones* in the repertoire
Day 0	4758	41	1.19%
Day 3	17333	160	2.06%
Day 7	19808	500	20.3%
Day 10	19596	1186	68.0%
CD154+	8596	1049	84.0%

* selected at day10

Figure S4: Identification of antigen responsive T cell clones reproduced in an additional donor.
A) Schematic representation of the in vitro culture system used to test donor 2. The same culture conditions where applied as described in Fig.1 a part for the final step which consisted in the isolation of CD154+ cells using magnetic beads enrichment. B-C) MA plots showing the differential expansion of individual TCR clones when comparing the day 0 and day 10 repertoire (B) and the day 10 and the CD154+ enriched fraction repertoire (C). D) Cross-table showing the overlap between clones defined as Differentially Over-Expanded (DOE), Not Differentially Expanded (NDE) or Differentially Under-Expanded (DUE) in the day 10 to day 0 repertoire comparison, and clones defined as Differentially Over-Represented (DOR), Not Differentially Represented (NDR) or Differentially Under-represented (DUR) clones in the enriched CD154+ fraction to day 10 repertoire comparison.
ND = Not Detected. E) Table reporting the number and impact (i.e. cumulative frequency) of the TCR clones selected as Differentially Over-Expanded (DOE) at day 10 at different timepoints during the 10 days stimulation culture.

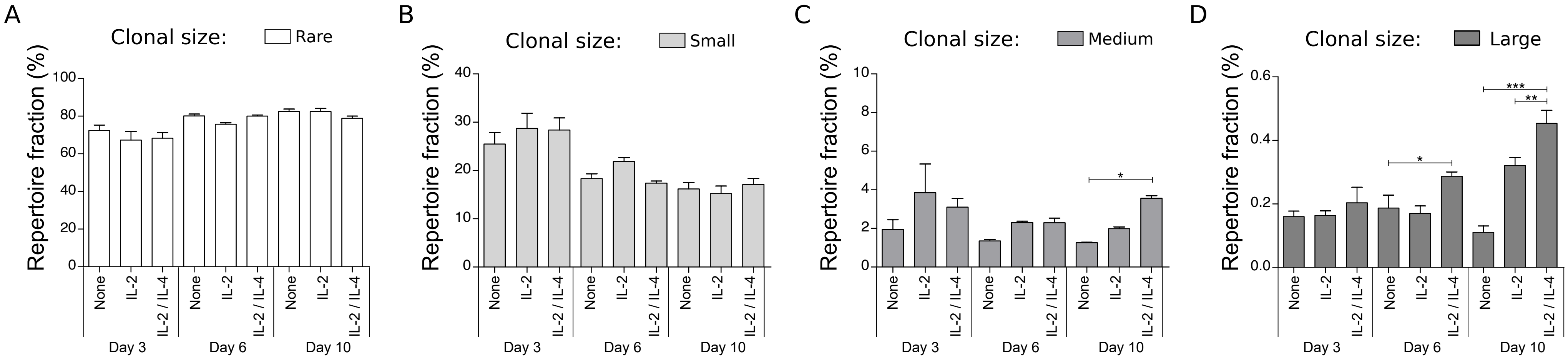

Figure S5: Clonal size distribution after in vitro antigen stimulation in different cytokine milieus.
Clonal size distribution in the TCR repertoire obtained at different time points during in vitro antigen stimulation assay in different cytokine environments. Each histogram depicts the percentage of the repertoire occupied by (A) rare (single UMI-count clones, clonal frequency (c.f.) = 0.004% in our dataset), (A) small (0.004% < c.f. < 0.1%), (C) medium (0.1% ≤ c.f. <0.5%) or (D) large (c.f. ≥ 0.5%) TCR clones. Bars show mean and standard deviation (*** p ≤ 0.0.1, ** p ≤ 0.01, * p ≤ 0.05 using one-way ANOVA followed by Bonferroni's multiple comparison posttest).

Peptide	Virus*	Protein	Fragment	Sequence
**Peptide 1**	EBV	EBNA1	475-489	NPKFENIAEGLRALL
**Peptide 2**	EBV	EBNA1	485-499	LRALLARSHVERTTD
**Peptide 3**	EBV	EBNA1	515-528	TSLYNLRRGTALAI
**Peptide 4**	EBV	EBNA1	529-543	PQCRLTPLSRLPFGM
**Peptide 5**	EBV	EBNA3C	961-986	AQEILSDNSEISVFPK
**Peptide 6**	Flu A	HA	97-112	CYPYDVPDYASLRSLV
**Peptide 7**	Flu A	HA	306-318	PKYVKQNTLKLAT
**Peptide 8**	Flu A	HA	417-432	KIDLWSYNAELLVALE
**Peptide 9**	CMV	pp65	41-55	LLQTGIHVRVSQPSL
**Peptide 10**	CMV	pp65	489-503	AGILARNLVPMVATV

*EBV = Epsten-Barr virus, CMV = Cytomegalovirus and Flu A = Influenza A virus.

Table S1: Peptides used for the in vitro T cell stimulation assays

		Cluster no.									
		**1**	**2**	**3**	**4**	**5**	**6**	**7**	**8**	**9**	**10**
**Day0 vs Day3**	**DOE**								1		
	**NDE**	13650	399	910	1052	11130	704	710	898	471	802
	**DUE**										
**Day5 vs Day0**	**DOE**		24						1		
	**NDE**	1129	756	1059	13368	11130	704	710	898	298	802
	**DUE**										
**Day10 vs Day0**	**DOE**		134	388						24	
	**NDE**	986	608	4507	1040	11123	696	683	505	408	799
	**DUE**					7	8	27			3

Table S2: Cluster assignment of differentially expanding TCR clones.
Distribution of the edgeR-selected Differentially Over-Expanded (DOE), Not-Differentially Expanded (NDE) and Differentially Under-Expanded (DUE) TCR clones at day 3, 5 and 10 after stimulation over the 10 expansion pattern clusters identified using K-mean clustering.

Repertoire	Total clones in the repertoire	Number of DOE clones* in the repertoire	Impact of DOE clones* in the repertoire
Day 0	43119	136	0.83%
Day 3	39566	215	1.87%
Day 5	40260	337	7.61%
Day 10	20474	548	61.4%
CD154+CD25+	5019	474	86.9%

* selected at day10

Table S3: Impact of the day 10-selected DOE clones on the repertoire during the T cell stimulation assay.
Table reporting the number and impact (i.e. cumulative frequency) of the TCR clones selected as Differentially Over-Expanded (DOE) at day 10 at different timepoints during the 10 days stimulation assay culture
